# Supplementary material for: The role of enhanced velocity shears in rapid ocean cooling during Super Typhoon Nepartak 2016
Source: Nat Commun. 2019 Apr 9;10:1627. doi: 10.1038/s41467-019-09574-3 (PMC6456504; doi:10.1038/s41467-019-09574-3)
Supplement: Supplementary file 2 — Description of Additional Supplementary Files [file 41467_2019_9574_MOESM2_ESM.pdf]

Description of Additional Supplementary Files

Supplementary Video 1:

A stand-alone time lapse camera that was mounted on NTU2 recorded sea surface images at 1-min intervals, which capture waves and air-sea interface during Super Typhoon Nepartak. Reference images of normal sea state one month after Nepartak are also demonstrated.
