# Supplementary material for: The role of enhanced velocity shears in rapid ocean cooling during Super Typhoon Nepartak 2016
Source: Nat Commun. 2019 Apr 9;10:1627. doi: 10.1038/s41467-019-09574-3 (PMC6456504; doi:10.1038/s41467-019-09574-3)
Supplement: Supplementary file 3 — Supplementary Information [file 41467_2019_9574_MOESM3_ESM.pdf]

Supplementary Information for:

The role of enhanced velocity shears in rapid ocean cooling during Super Typhoon Nepartak 2016

Yang et al.

Institute of Oceanography, National Taiwan University

No. 1, Sec. 4, Roosevelt Rd., Taipei 10617, Taiwan

Correspondence to: Y. J. Yang, Email: yjyang67@ntu.edu.tw

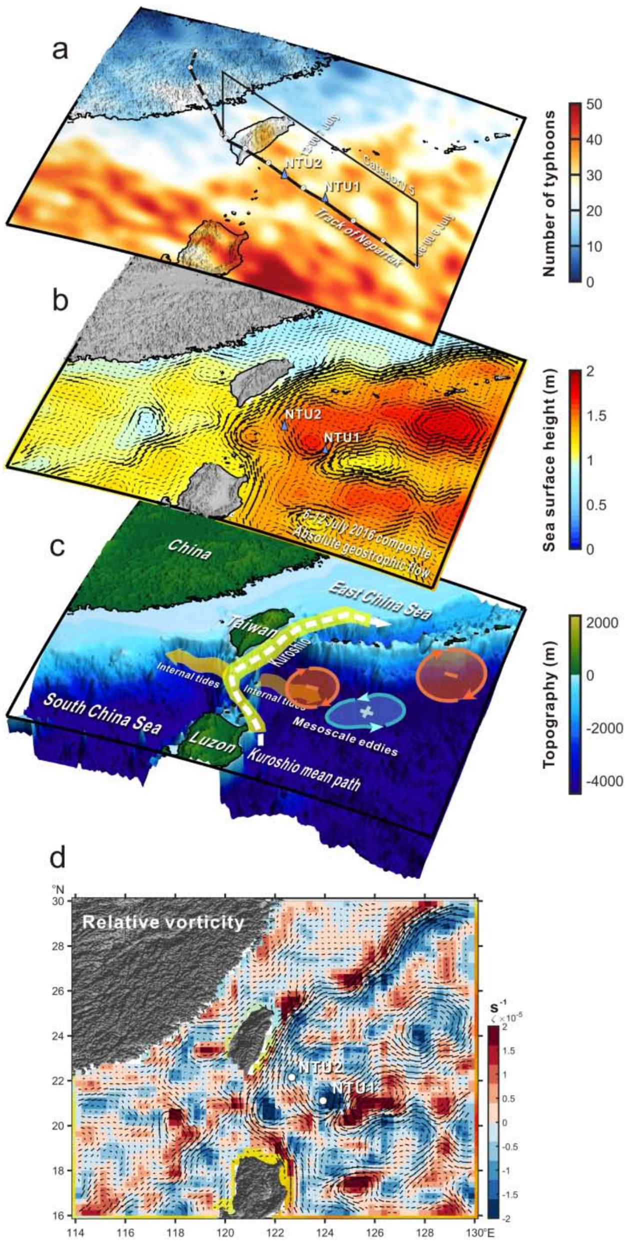

Supplementary Figure 1. Statistics of typhoons and oceanic background environment during July 2016. a Numbers of typhoon passage in each 0.5°×0.5° grid (color shading) during 1951 and 2017, overlaid with Super Typhoon Nepartak's track (white line) and the two buoy's location (blue triangle). **b** Composite satellite sea surface height (color shading) and associated absolute geostrophic currents (arrows) during 6-12 July 2016 obtained from AVISO. **c** Schematic diagram showing the Kuroshio, mesoscale eddies, and internal tides off Taiwan in the western North Pacific. **d** Relative vorticity (color shading) of the geostrophic flow field in **b**.

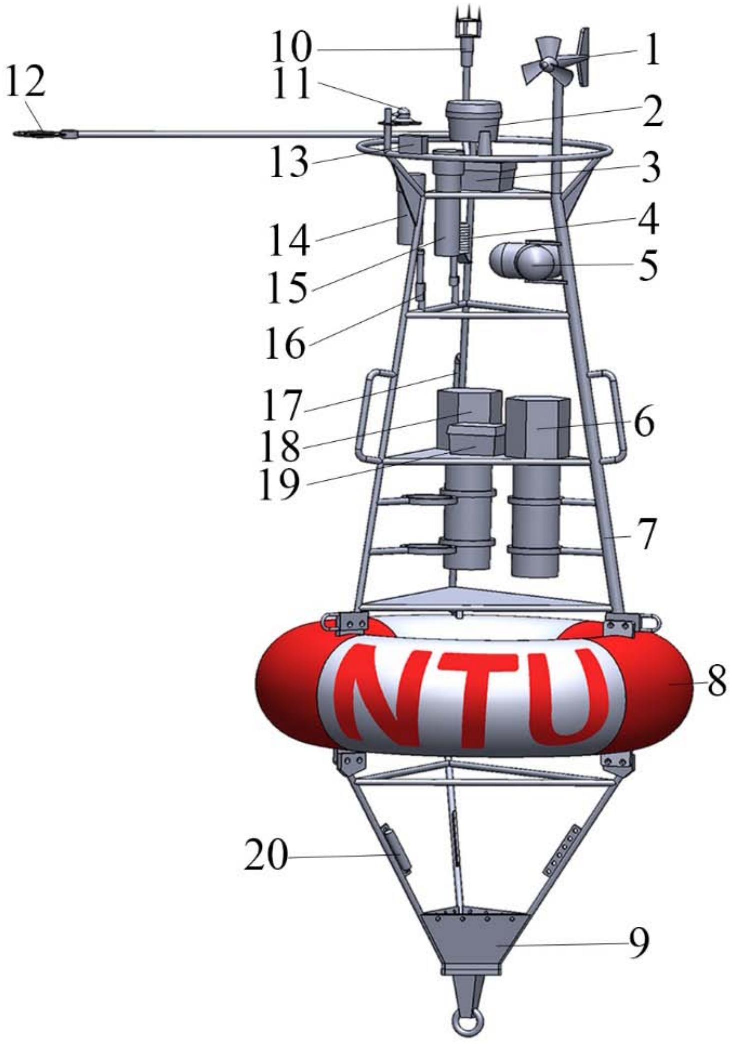

Supplementary Figure 2. Schematic showing relative locations of instruments and meteorological sensors on the surface buoy: 1-propeller anemometer, **2**-solar marine navigation light, **3**-Iridium satellite antenna, **4**-air temperature and relative humidity probes, **5**-radar reflector, **6**-control and communication unit, **7**-stainless steel tower, **8**-fiberglass buoy, **9**-bride, **10**-ultrasound anemometer, **11**-pyrometer, **12**-net radiometer, **13**-time lapse camera, **14**-Iridium GPS beacon, **15**-rain gauge, **16**-UHF antenna, **17**-barometer, **18**-meteorological data acquisition unit, **19**-digital compass, and **20**-temperature sensor.

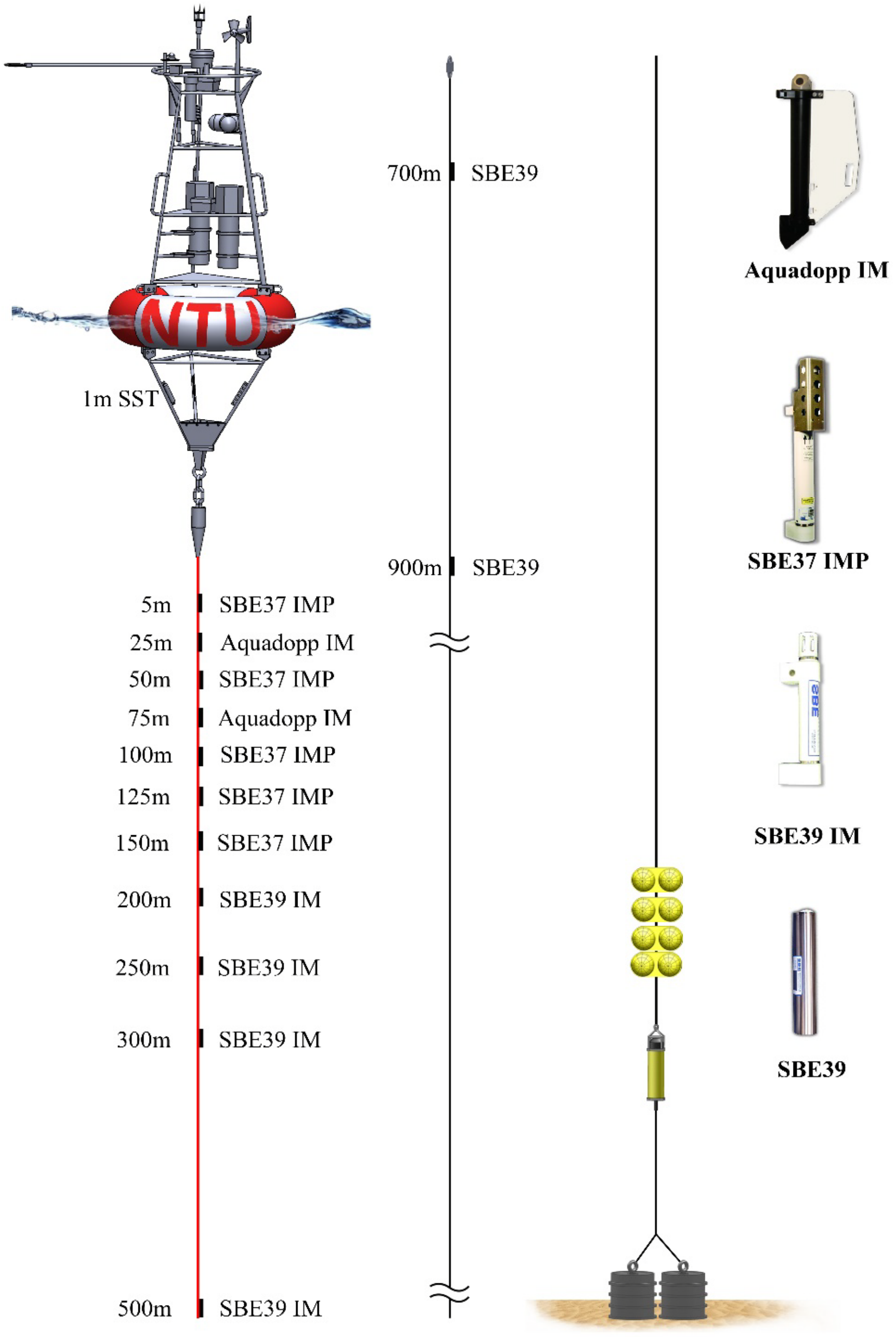

Supplementary Figure 3. Schematic showing the mooring diagram and relative depths of subsurface instruments of the buoy. Aquadopp IM is current meter with inductive modem; SBE37 IMP is CTD with inductive modem and pump; SBE39 IM and SBE 39 are temperature-pressure recorder with and without inductive modem, respectively.
